# Fibroblast-Generated Extracellular Matrix Guides Anastomosis during Wound Healing in an Engineered Lymphatic Skin Flap

**DOI:** 10.3390/bioengineering10020149

**Published:** 2023-01-22

**Authors:** Alvis Chiu, Wenkai Jia, Yumeng Sun, Jeremy Goldman, Feng Zhao

**Affiliations:** 1 Stem Cell and Tissue Engineering Lab, Department of Biomedical Engineering, College of Engineering, Texas A&M University, College Station, TX 77843, USA; 2 Vascular Materials Lab, Department of Biomedical Engineering, College of Engineering, Michigan Technological University, Houghton, MI 49931, USA

**Keywords:** lymphatic, extracellular matrix, decellularized matrix, tissue engineering, in vitro model, self-assembled vessels

## Abstract

A healthy lymphatic system is required to return excess interstitial fluid back to the venous circulation. However, up to 49% of breast cancer survivors eventually develop breast cancer-related lymphedema due to lymphatic injuries from lymph node dissections or biopsies performed to treat cancer. While early-stage lymphedema can be ameliorated by manual lymph drainage, no cure exists for late-stage lymphedema when lymph vessels become completely dysfunctional. A viable late-stage treatment is the autotransplantation of functional lymphatic vessels. Here we report on a novel engineered lymphatic flap that may eventually replace the skin flaps used in vascularized lymph vessel transfers. The engineered flap mimics the lymphatic and dermal compartments of the skin by guiding multi-layered tissue organization of mesenchymal stem cells and lymphatic endothelial cells with an aligned decellularized fibroblast matrix. The construct was tested in a novel bilayered wound healing model and implanted into athymic nude rats. The in vitro model demonstrated capillary invasion into the wound gaps and deposition of extracellular matrix fibers, which may guide anastomosis and vascular integration of the graft during wound healing. The construct successfully anastomosed in vivo, forming chimeric vessels of human and rat cells. Overall, our flap replacement has high potential for treating lymphedema.

## 1. Introduction

It is estimated that 287,850 women developed breast cancer in 2022 in the US, with 10% not surviving [1]. Among the survivors, 20% [2] to 49% [3] will develop breast cancer-related lymphedema (BCRL), leading to heaviness, numbness, and tightness in the affected limb. This disease not only drastically lowers the patient’s quality of life [1,4], but also creates an alarming healthcare burden for mental health services, disease monitoring, and disease treatment [5]. BCRL is a frequent consequence of axillary lymph node dissection or sentinel lymph node biopsy in cancer patients, which can interrupt the resorption of excess interstitial fluid of the arm and its transport back to the venous circulation. This permanently decreases lymph transport capacity and often causes fluid buildup, painful arm swelling and susceptibility to infections. Currently, there is no cure for BCRL, only preventative or palliative treatments. During the early stages of lymphedema, before the onset of fibrosis due to the accumulation of protein and lipids, swelling can be improved by manually compressing the limb using garments, bandages, or intermittent pneumatic compression therapy [6,7,8]. Unfortunately, these modalities require intense lifelong effort and are ineffective for advanced fibrotic limbs where the tissue has already irreversibly remodeled from the excess interstitial fluid [6,9].

Vascularized lymph node transfer is the most commonly used procedure for reducing lymphedema in advanced lymphedema patients [10]. It is accomplished by transplanting skin or adipose flaps containing functional lymph nodes harvested from a healthy donor site [11,12]. The implanted lymph nodes spontaneously anastomose with recipient site lymphatic and venous vessels and drain lymph fluid from those respective systems [11,12,13]. However, this procedure has an 18% chance of causing seroma [14,15], and harvesting lymph nodes from the preferred groin donor site can create chronic lymphedema [14,16]. Moreover, recent evidence has shown that the lymph nodes were actually irrelevant to the lymphedema reduction, as the therapeutic agents of lymph node transfers were in fact the surrounding lymphatic vessels [17]. In 2016, Koshima et al. completed the first pilot study of vascularized lymph vessel transfer (VLVT), which aimed to minimize donor site morbidity by not sacrificing lymph nodes [13,18]. The effectiveness of this treatment has been proven by multiple clinical trials that showed relief of symptoms, weaning of compression garments, and improvement of quality of life [17,18,19,20,21]. Although donor site lymphedema has not been reported for VLVT [18], donor site availability still significantly limits its application. Therefore, there is a crucial need for skin and adipose flap substitutes.

Various novel therapeutics have been proposed to induce the growth of new lymphatic vessels across the dissected areas to improve lymph drainage. Vascular endothelial growth factor-C therapies have shown effectiveness in reducing lymphedema by directly signaling lymphatic endothelial cells (LECs) to sprout more capillaries from existing capillaries [22,23]. Nanofibrous collagen scaffolds have been utilized to create temporary bridges that direct interstitial fluid flow over the damaged areas to guide lymphangiogenesis [24]. Mesenchymal stem cell (MSC) therapy reduced lymphedema by secreting lymphangiogenic and immunomodulatory factors that augment the wound microenvironment [25]. Although these prolymphangiogenic therapies have achieved success in reducing acute lymphedema in animal models, long-term functional lymphangiogenesis is inhibited by the chronic inflammation and fibrosis present in mild to severe lymphedema [9,25,26]. Therefore, these approaches may be inadequate for treating chronic or severe cases.

Since stimulating peri-wound lymphangiogenesis to restore lymphatic continuity may be challenging for advanced lymphedema patients, the implantation of tissue engineered lymphatic vessels may serve as a more effective treatment modality. LECs can self-assemble into lymphatic microvascular networks in the presence of various supporting cells. Co-culturing LECs with adipose-derived MSCs formed stable networks for up to 4 weeks [27]. Similarly, 3D lymphatic networks with native ultrastructure have been generated by seeding LECs on fibroblast sheets [28]. More recently, LECs co-cocultured with fibroblasts on a collagen sheet were shown to anastomose with host lymphatics in a mouse model [29]. However, the aforementioned approaches lacked guidance cues, so the lymphatic vessels formed were randomly oriented, which made them deficient in the natural lymphatic vessel alignment (axiality) that facilitates unidirectional lymph flow in native tissues [20]. Decellularized adipose tissue has also been used as a scaffold to generate lymphatic networks with anastomosis capacity [30]. While decellularization may preserve the lymphatic axiality of the tissue as newly seeded LECs colonize existing vessel channels, decellularized scaffolds suffer from problems of donor scarcity, host responses, and pathogen transfers when allogeneic or xenogeneic tissues are used [31]. Compared with reconstituted or decellularized scaffolds, cell-derived extracellular matrix (ECM) has several advantages: (1) Sterile culture conditions eliminate pathogen contamination risks. (2) Cell-derived ECM can be engineered with controlled topography and porosity to guide lymph axiality. (3) Cell-derived ECM modulates host immune responses, reducing fibrosis [31]. Therefore, cell-derived ECM offers a promising alternative to scaffolds derived from natural tissues.

The objective of this study was to utilize cell-derived ECM to develop an engineered lymphatic flap that mimics the dermal and lymphatic components of a native ultra-thin skin flap used in VLVT. The construct consists of three layers: (1) decellularized fibroblast ECM, (2) human MSCs, and (3) self-assembled lymphatic capillaries. During VLVT, due to the bulk of the skin flap, a pedicled artery and vein that spans the graft needs to be surgically anastomosed to perfuse the graft [18]. We expect that, by eliminating the unnecessary epidermal, blood, and subcutaneous components and only keeping the relevant cells for lymphedema reduction, our construct may function without a dedicated blood supply. The construct is created on a nanograted polydimethylsiloxane (PDMS) substrate that guides fibroblast and fibroblast-secreted ECM alignment, which further directs MSC alignment, and in turn, LEC alignment. Aligned LECs form unidirectional capillaries, mimicking the native lymphatic axiality of skin flaps [20,32,33]. They also have the cellular and ultrastructural features of native human dermal lymphatic capillaries [28]. Upon implantation, MSCs reduce inflammation [34], secrete lymphangiogenic factors [35], and serve as supporting cells for the lymphatic capillaries [36]. The fibroblast-derived ECM itself also promotes wound healing, reduces fibrosis, and provides mechanical support for the flap [36,37]. This approach can incorporate autologous cells, thereby eliminating the use of allogeneic or xenogeneic tissues while conserving lymph axiality. This skin flap replacement has the potential to increase graft availability and reduce lymphatic damage-related surgical procedures and complications.

## 2. Materials & Methods

### 2.1. Cell Culture

All cells used were obtained from commercial sources. Clonetics™ Dermal Lymphatic Microvascular Endothelial Cells (LECs) and Poietics^TM^ Normal Human Bone Marrow Derived Mesenchymal Stem Cells (MSCs) were obtained from Lonza, Basel, Switzerland. Normal Human Adult Primary Dermal Fibroblasts (HDFs) were obtained from American Type Culture Collection, Manassas, VA, USA. LECs at passage 5, were cultured in endothelial growth medium 2 (EGM-2, Lonza). Passage 7 HDFs were cultured in fibroblast growth media consisting of 60% Dulbecco’s Modified Eagle Medium, 20% F-12, 20% fetal bovine serum (FBS) and 1% penicillin/streptomycin. Passage 4 MSCs were cultured in Minimum Essential Medium α with 20% FBS, 1% penicillin/streptomycin, and 1% L-glutamine (Thermo Fisher Scientific, Waltham, MA, USA). All cells were cultured in a humidified, 37 °C, 5% CO_2_ incubator.

### 2.2. Wound Healing Model

**Figure 1:**
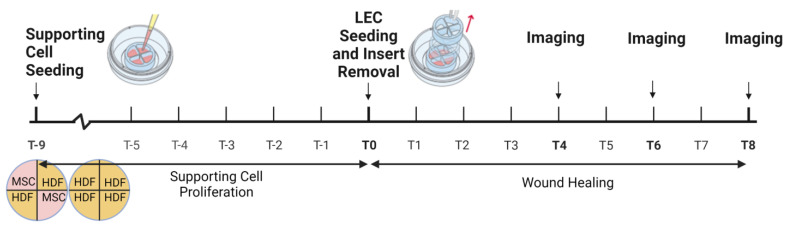
Timeline of in vitro wound healing model. The culture-insert is first attached to a well plate. The supporting cells, human dermal fibroblasts (HDFs) and mesenchymal stem cells (MSCs), are cultured inside for 9 days in either the MSC-HDF or HDF-HDF configuration. In MSC-HDF, the supporting cells alternate between MSCs and HDFs, whereas HDF-HDF only contain HDFs. Subsequently, lymphatic endothelial cells (LECs) are seeded on top for 10 h of vessel assembly, or lymphangiogenesis, before culture-insert removal. Three inserts were used for each configuration at each imaging timepoint.

The wound healing model used 4 well culture-inserts (Ibidi, Fitchburg, WI, USA) to create bilayered lymphvascularized tissues separated by wound gaps. Each insert is cylindrical with an outer diameter of 17 mm and inner diameter of 13 mm and has 4 hollow quadrants dividing the center into 4 wells each with a growth area of 0.35 cm^2^ per well. The quadrants are separated by walls that create a 500 ± 100 µm cell-free gap between each quadrant. The inserts were placed into 12-well plates. MSCs were seeded at 5000 cells/cm^2^ and HDFs at 3000 cells/cm^2^ in their respective media in either the MSC-HDF or HDF-HDF configuration (Figure 1). These seeding densities were optimized to make both supporting cell types reach confluency on the same day while minimizing aggregate formation. In MSC-HDF, each wound gap is flanked by one MSC side and one HDF side. For HDF-HDF, both sides are HDF. Four wound gaps were created using one insert for a total of 12 gaps per timepoint for each configuration. Cell culture media was changed every 3 days until both MSCs and HDFs reached confluency after 9 days of culture. LECs at 20,000 cells/cm^2^ were seeded on top of the basal cells after basal cell confluency. The culture-insert was removed 10 h after LEC seeding to allow for 10 h of lymphangiogenesis. The culture media was then switched to a mixture of 50% fibroblast growth media and 50% EGM-2 and was changed every 2 days. Wound gaps were examined 4, 6, and 8 days after insert removal.

### 2.3. Immunofluorescence Staining

Skin explants of implanted engineered flaps and cell cultures of the wound healing model were fixed in 4% paraformaldehyde in PBS containing calcium and magnesium for 20 min and blocked with a solution of 2% bovine serum albumin (Sigma-Aldrich, St. Louis, MO, USA). All antibodies were diluted in blocking solution and applied overnight on a rocker at 4 °C. For the wound healing assay, LECs were stained using anti-CD31 antibodies, and ECM was stained using anti-collagen I antibodies. For the animal tissue sectioning, LECs were stained for podoplanin (PDPN). To distinguish between rat and human LECs, human nuclear antigen (HNA) was targeted. All IgG secondary antibodies (Alexa Fluor 488, 568, and 647 goat anti-rabbit and goat anti-mouse) were purchased from Thermo Fisher Scientific diluted 1:500 in blocking solution. All imaging was done on a Zeiss Observer 3 microscope using either a Zeiss 20× air or 40× water objective and an Axiocam 503 mono camera (Zeiss, Oberkochen, Germany).

### 2.4. Quantitating Endothelial Invasion

Images of the wound healing model were taken according to the aforementioned method and stitched using the MosaicJ plugin for the open-source image processing program ImageJ. A 600 × 6500 µm rectangle containing the original wound gap was selected for analysis for vertical wound gaps. The dimensions of the analysis area were flipped for horizontal wound gaps. The exact position of the analysis rectangle was first determined by macroscopically locating the wound gap and then placing the box around the middle of the two sides of invading vessels. The angle of the box was adjusted accordingly as the culture-inserts were placed by hand. Vessel area was calculated by thresholding CD31 signal and using the analyze particles command. The area was measured in triplicates by two researchers independently.

### 2.5. Lymphatic Skin Flap Replacement Fabrication

The engineered flap was fabricated following our published protocol [32,38]. Briefly, PDMS substrates were cast from molds with 350 nm grating width and 130 nm grating depth and coated with collagen I. HDFs were seeded on top and cultured for 3 weeks in fibroblast growth media with a media change every 3 days. This produces uniform 70 μm-thick cell sheets [38], which were gently peeled off and decellularized with 0.5% sodium dodecyl sulfate, 10 mM Tris (Bio-rad Laboratories, Hercules, CA, USA), and 25 mM Ethylenediaminetetraacetic acid (Sigma-Aldrich) solution for 15 min. Afterwards, the ECM sheets were thoroughly washed with PBS and incubated in culture media for 48 h. The ECM sheets produced by our decellularization protocol retain collagen I, elastin, and fibronectin fibers and have an elastic modulus of around 250 Pa and a viscous modulus of 300 Pa [38]. The fibers are uniformly aligned with a dimeter of 78 ± 9 nm [38]. MSCs were seeded at 10,000 cells/cm^2^ on the decellularized ECM sheet and cultured under hypoxia (2% O_2_) for 7 days. Subsequently, LECs were seeded at 20,000 cells/cm^2^ on top of the MSC/ECM constructs. The co-cultures were maintained at 20% O_2_ for up to 7 days in EGM-2 with a media change every 2 days. This method has shown to generate vessel networks with a mean vessel diameter of 11 µm, a mean vessel length of 220 µm, an intercapillary distance of 19 µm, and a 14% area vessel coverage [32,33].

### 2.6. Animal Model

All animal experiments were performed following protocols approved by the institutional committee for animal use and care regulations at Michigan Technological University. Athymic Rowett nude (RNU) rats (male, 6–8 weeks old, 200–250 g weight) were purchased from Charles River Laboratories (Wilmington, MA, USA). Rats were anesthetized with isoflurane and their backs were shaved with clippers. A small incision was made on the rat dorsum to expose the subcutaneous space, in which a stack of three 2 cm^2^ disks of the engineered lymphatic flap was implanted. Incisions were closed with suture clips. The rats were euthanized at day 7 post implantation and the skin was harvested from the back that contained the engineered lymphatics. A region of skin from another random area from the back was also harvested to serve as a control. A total of 2 samples were harvested from each of 3 rats. The skin explants were stained as mentioned above.

### 2.7. Statistical Analysis

CD31 coverage data from each timepoint (day 4, 6, 8) for MSC-HDF and HDF-MDF gaps were compared using one-way ANOVA and Tukey’s post hoc test on GraphPad Prism. The results were reported as mean ± standard deviation. Results were considered statistically significant for *p* ≤ 0.05. Datapoints from detaching or aggregating tissues were excluded to prevent the detachment or aggregation from confounding the results.

## 3. Results

### 3.1. Flap Anastomoses with Self-Assembled Lymphatic Capillaries

**Figure 2:**
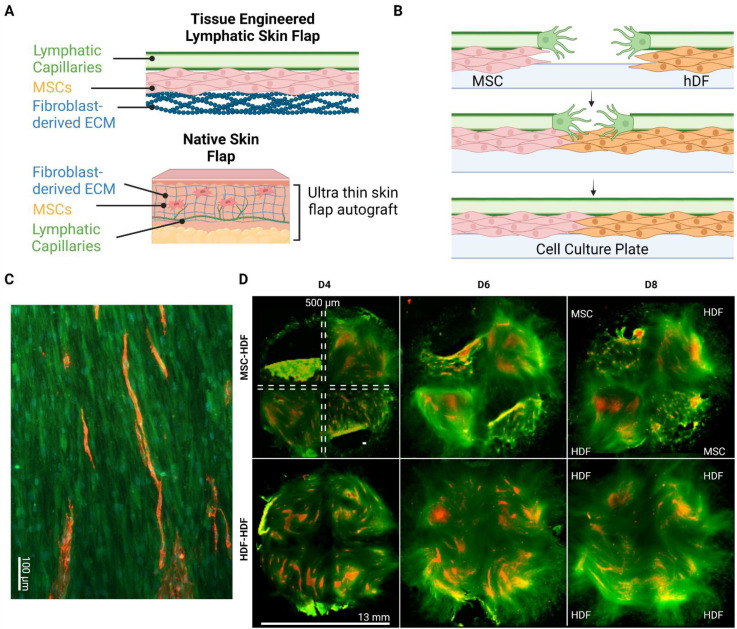
Engineered lymphatic flap and wound healing model. (**A**) Composition of engineered lymphatic flap. The three layers of the engineered flap (lymphatic capillaries, MSCs, and HDF extracellular matrix (ECM)) mimics the ultra-thin skin flaps used in vascularized lymph vessel transfer (VLVT). (**B**) Mechanism of bilayered wound healing assay. Capillary formation and sprouting is limited by the basal ECM. Supporting cell proliferation and ECM secretion allows lymphangiogenesis over the inhospitable culture plate substrate. (**C**) Immunostaining of engineered flap. LECs (Red). Collagen I (Green). Cell Nuclei (Blue). Scale bar: 100 µm. (**D**) Natural collagen swirls forming in the process of wound healing. Lymphatic capillaries colocalized with these ECM patterns. Wound gaps with detached tissues were excluded for analysis. LECs (red) Collagen I (Green). Wound gap: 500 µm. Scale bar: 13 mm.

**Figure 3:**
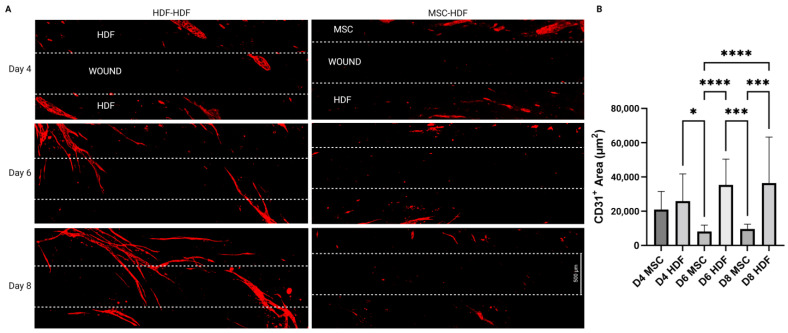
Time lapse images of capillary invasion into the wound gap after a 10-h lymphangiogenic period. (**A**) After basal cell coverage of the wound gap at day 4, self-assembled capillaries started protruding into the filled gap over the basal cells in HDF/HDF interfaces. Capillaries from opposing sides pointed towards each other. Capillary invasion was not observed in MSC-HDF interfaces. (**B**) Capillary coverage is present in wound gaps between HDF and either MSC or HDF. HDF-HDF had significantly more vessel invasion than MSC-HDF on day 6 and 8. Sample sizes: D4 MSC (*n* = 8) D4 HDF (*n* = 10), D6 MSC (*n* = 8), D6 HDF (*n* = 12), D8 MSC (*n* = 8), D8 HDF (*n* = 11). * *p* ≤ 0.05, *** *p* ≤ 0.001, **** *p* ≤ 0.0001. Scale bar: 500 µm.

**Figure 4:**
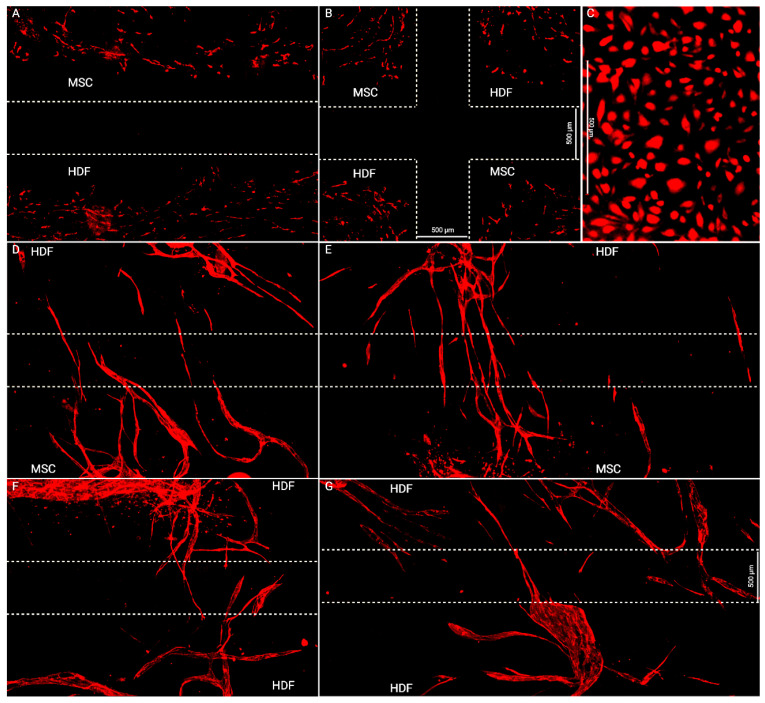
Images of capillary invasion into the wound gap after a 48-h lymphangiogenic period. (**A**,**B**) Day 4. Supporting cells have closed the wound gap. Some capillaries have formed, but not yet invaded the wound gap. Capillaries were still short and immature. (**C**) LECs fail to form capillaries when seeded directly on the culture dish. Capillary invasion over wound gaps must therefore be over basal cells. (**D**–**G**) Day 8. Capillaries have invaded the wound gap. MSC-HDF gaps had more connecting capillaries than HDF-HDF. Scale bar: 500 µm.

**Figure 5:**
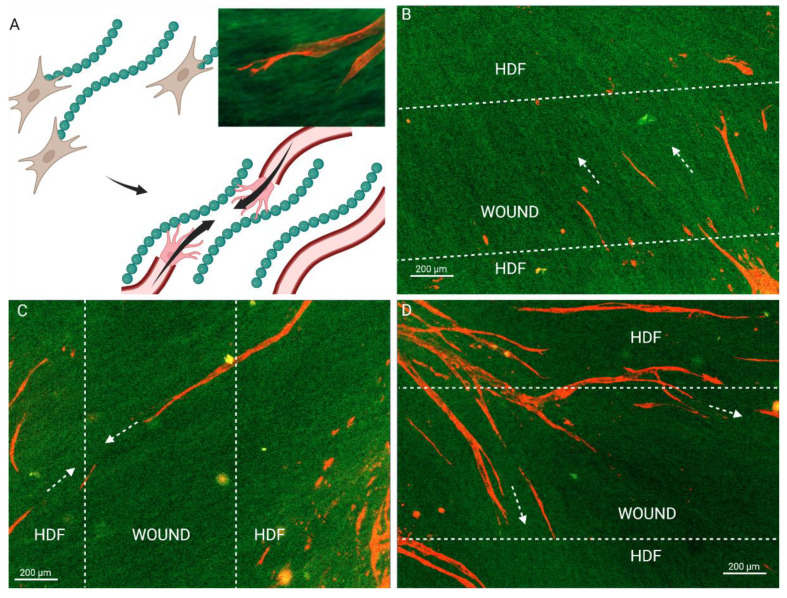
Fibroblasts guide capillary integration through natural collagen I alignment during in vitro graft integration. (**A**) ECM-mediated anastomosis model, where end-to-end anastomosis of capillaries from opposing wound edges are guided by collagen tracks. (**B**–**D**) Fibroblast proliferation and migration patterns in the wound gap leave behind collagen tracks that the capillaries follow. Arrows mark the direction of the collagen fibers as well as capillary invasion. LECs (red) Collagen I (Green). Scale bar: 200 µm.

To investigate the anastomosis capacity of the engineered flap with host lymphatics, an in vitro model was designed to replicate the integration of the flap with the host. Basal cells (MSCs in the present case) and then LECs were seeded on HDF-derived ECM to simulate flap transplantation (Figure 2A). The basal cells and LECs were also seeded directly on the culture plates (Figure 2B–left side), where LECs underwent lymphangiogenesis and formed capillaries on top of the basal cells on both sides of a 500 µm wide wound gap (Figure 3A). LECs failed to form capillaries when seeded alone on the culture plate (Figure 4C), indicating the necessity of basal supporting cells and their deposited ECM for lymphangiogenesis. Thus, the bilayered co-culture setup created a network of self-assembled capillaries on top of a traditional scratch assay (Figure 2B), where capillary sprouting was both enabled and confined by basal cell expansion. The basal cell layer beneath the LECs modeled two different tissues. LEC/MSC represented the lymphatic flap, as the engineered flap is built upon MSCs (Figure 2C). LEC/HDF modeled the host recipient site as the fibroblasts recapitulated a dermal organoid. In either LEC/MSC or LEC/HDF, the wound-mimicking gap fully closed after 4 days, as shown by complete collagen coverage of the gap (Figure 2D). Capillaries on the wound edge then sprouted over the basal cells into the wound gap (Figure 3A). Once inside the gap, the capillaries exhibited minimal branching and a straight orientation. HDF-HDF interfaces had significantly more capillary coverage at day 6 and 8 compared to MSC-HDF (Figure 3B). The invasion of capillaries in HDF-HDF into the wound gap generally followed collagen I fibers generated by the migration and proliferation patterns of the basal cells (Figure 2D and Figure 5), and capillaries from opposing sides migrated towards each other (Figure 3A). In contrast to the results of HDF-HDF, capillaries in MSC-HDF underwent pruning and regression from day 4 to 8, resulting in a decrease of capillary coverage across the gap (Figure 2D and Figure 3A).

The unexpected lack of capillary coverage in MSC-HDF and the guiding effect of collagen fibers in HDF-HDF led us to investigate the role of basal cell-deposited ECM in lymphangiogenesis. The original protocol was modified by increasing the lymphangiogenic period between LEC seeding and culture insert removal from 10 h to 48 h to allow for more buildup of the MSC layer. While this increased capillary invasion and produced capillaries that crossed over the entire wound gap in both MSC-HDF and HDF-HDF (Figure 4), this also exacerbated the aggregation of MSCs, causing capillaries to be pulled into aggregates (Figure S1), so a statistical analysis could not be performed.

### 3.2. Engineered Flap Spontaneously Anastomoses with Rat Model

**Figure 6:**
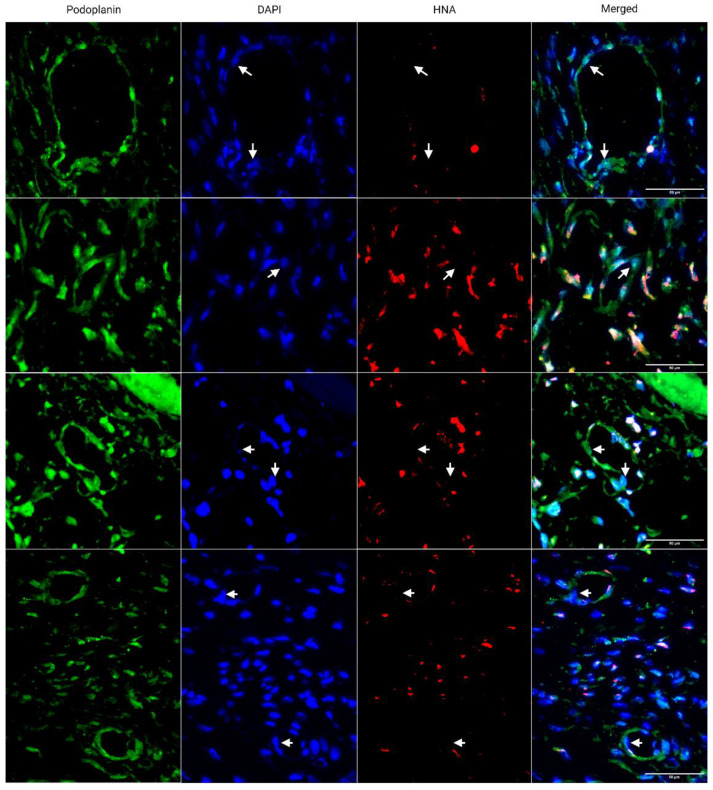
Immunostaining of implanted engineered lymphatic flap. Rat-human chimeric capillaries were found 7 days after subcutaneous implantation in athymic nude rats (*n* = 3). Rat, Human PDPN (green), HNA (red). Each row represents a different site of anastomosis. Arrows point to HNA^-^ LECs within HNA^+^ vessels. Scale bar: 50 µm.

To determine the anastomosis capacity of the engineered flap after implantation, the flap was subcutaneously implanted in athymic nude rats, which can prevent xenograft rejection. Seven days post implantation, chimeric capillaries made of rat and human LECs were observed (Figure 6). Human nuclear antigen (marker of human cells) negative rat LECs were found incorporated into human capillaries, indicating the fusion of implanted human capillaries with host rat capillaries. The sectioned lumens were also open.

## 4. Discussion

The supply of skin autografts for VLVT is limited. We have fabricated an engineered lymphatic flap replacement that is both scalable and personalizable to the patient. In vitro results showed that the proliferation of supporting cells in the engineered lymphatic flap paved the way for lymphangiogenesis, which leads to the connection of graft and host capillaries. Subcutaneous implantation also indicated that the engineered flap is capable of spontaneous anastomosis in vivo. Therefore, our construct has high potential for ameliorating advanced fibrotic lymphedema.

ECM deposition by graft and host cells plays an underappreciated role in graft vascular integration. MSC-HDF was expected to have more vessel invasion due to the chemotactic and lymphangiogenic effects of MSC-secreted paracrine factors [25]. The unexpectedly low levels of capillary invasion in MSC-HDF may therefore have been caused by insufficient matrix deposition over the wound gap by MSCs as HDFs proliferated and deposited matrix faster than MSCs (Figure 2D), which correlated with higher capillary invasion in HDF-HDF (Figure 3B). Extension of the lymphangiogenic period may have rescued the lack of capillary coverage of the MSC-HDF wound gaps (Figure 4) by allowing more accumulation of MSC-deposited ECM, which may have improved the quantity and quality of capillaries on the wound edges that participated in the invasion. The MSC layer may have also increased in thickness and cell density, which can contribute to the overall ECM deposition rate into the wound gap. In addition to providing a substrate for lymphangiogenesis, ECM can signal surface topographical cues known to guide cell alignment by localizing focal adhesions along grooves [39]. We previously found that aligned ECM nanofibrous scaffolds guided capillary alignment in a multi-layered vascular construct [32]. An aligned electrospun gelatin scaffold has also shown a similar ability to direct angiogenesis [40]. Coincidentally, thin oriented collagen fibers are also deposited during skin wound healing [41]. Therefore, the ECM fibers secreted by HDFs during wound healing may direct and promote capillary invasion much like an aligned scaffold would in addition to providing mechanical support to the wound. We posit that continuous ECM fibers from two sides of a wound facilitate capillary invasion and end-to-end anastomosis as capillaries from the two opposing sides follow the same fibers and come into contact (Figure 5). This may be a novel mechanism of wound healing and graft revascularization and integration. Further research into understanding the mechanisms of graft integration could improve the success rate of tissued engineered grafts.

Our model is in agreement with past wound healing models. The morphology of the invading capillaries from our system is comparable to that of animal wound healing and ex vivo models. Our 2D assay reflects cross sections of the healing fibrin clot of a full-thickness porcine wound model [42]. Similar to our results, the porcine capillaries extended from the granulation tissue into the provincial matrix by day 4 of wound repair. An explant model using artery and vein sections cultured 500~1000 µm apart reported similar anastomosis patterns [43]. A micropatterned substrate covered in thymosin β4-hydrogel was used to induce directed capillary sprouting between two explants, and the outgrowths connected the two parent explants by day 21. The guided vessels resembled the capillaries in Figure 4D–G, further supporting our ECM-guidance hypothesis. The initial progressive increase in capillary area coverage of HDF-HDF is also consistent with murine [44] and human [45] skin autograft revascularization, where the graft integration layer increases in vascular density after implantation. However, Tefft et al. [46] reported no capillary invasion in their in vitro wound healing model of granulation tissue formation. Their model was created by seeding fibroblasts and human umbilical vein endothelial cells in a 3D collagen and fibrin gel in a microfluidic chamber. An incision was made after 3 days of capillary assembly by stabbing the gel with a dissection knife. This model also achieved a wound closure time of 4 days. However, unlike our results, their blood capillaries remained at the periphery of the wound as fibroblasts filled in the wound with fibronectin and collagen III. This could be because the fibroblasts initially migrated circumferentially around the wound during wound contraction before migrating to the center of the void. This would have left collagen tracks tangential to the wound, leading capillaries to sprout around it.

Upon implantation, the engineered flap spontaneously connected with host lymphatics in a rat model (Figure 6). Spontaneous anastomosis has been demonstrated in clinical lymph-interpositional-flap transfers, where the lymph vessel stump of a flap is placed closely to those of a recipient site without microsurgical anastomosis [10,11]. This is believed to be the primary mechanism by which implanted flaps connect to and restore recipient site lymph flow. Compatibility of lymph axiality of the graft and the recipient site is critical for lymph flow restoration and reducing lymphedema development risk. Our engineered flap is designed with highly aligned capillaries, giving it high lymph axiality and therefore high potential to restore lymph flow after anastomosis. The ECM fibers in the flap may have also provided interstitial flow guidance for lymphangiogenesis similar to nanofibrillar collagen scaffolds [15].

As this study was a proof-of-concept study, there were several limitations. First, although capillaries spanning across the wound gap were observed, end-to-end anastomosis of capillaries originating from opposite side was not confirmed. Capillaries may have primarily sprouted from one side to the other, so cell origin will be clarified by labeling the LECs. Second, the identity of the supporting cells was not verified. The cells covering the wound gap may have been mostly HDFs, as they proliferated faster. Aggregation was also prevalent when culture time increased in the MSC tissue because of the difficulties in using a tri-culture. We previously found that the optimal culture time for lymphangiogenesis was 7 days of co-culture with MSCs [32], but this caused aggregation and detachment in the model. While we minimized aggregation, viable wound gap samples still decreased from 12 to 8 in MSC-HDF and from 12 to 11 in HDF-HDF. However, this should not have affected the results as wound interfaces with detaching sides were excluded. The peeling and aggregation may have been caused by cellular detachment from the culture plate caused by the removal of the culture-inserts. In addition, live cell tracking can investigate the formation process of oriented collagen I fibers, and disruption of aligned collagen formation is needed to prove its role in capillary invasion guidance. This may be achieved by seeding basal cells in the wound gap to induce contact inhibition on peri-wound basal cells while still maintaining a basal cell layer for capillaries to invade over.

## 5. Conclusions

We engineered a lymphatic flap that is designed to replace skin flaps in VLVT. Using the fabrication techniques for the engineered flap, we developed a novel wound healing assay that reproduces the process of wound closure and graft integration. This model has revealed that collagen fibers deposited during wound healing may guide angiogenesis, facilitate anastomosis, and graft perfusion. Understanding the mechanism of graft integration will improve the success rate of transplants and engineered tissue constructs. The engineered lymphatic flap itself is capable of spontaneous connection with host lymphatics. Our findings propose a potential novel therapeutic for treating lymphedema patients with dysfunctional lymphatic vessels by increasing lymph drainage to the venous and lymphatic systems.

## Data Availability

The data presented in this study are available in the article and Supplementary Materials.

## Supplementary Materials

The following supporting information can be downloaded at: https://www.mdpi.com/article/10.3390/bioengineering10020149/s1, Figure S1: Aggregation in wound healing model at day 10. The 10-hour lymphangiogenic period was extended to 48 hours, resulting in thickened basal cell layer and more complete tube formation. However, basal cell contraction pulled the surface capillaries inward, sometimes over the wound gaps.
