# Supplementary material for: Fibroblast-Generated Extracellular Matrix Guides Anastomosis during Wound Healing in an Engineered Lymphatic Skin Flap
Source: Bioengineering (Basel). 2023 Jan 22;10(2):149. doi: 10.3390/bioengineering10020149 (PMC9952048; doi:10.3390/bioengineering10020149)
Supplement: Supplementary file 1 [file bioengineering-10-00149-s001.zip › bioengineering-2135322-SI.pdf]

bioengineering

Article

Fibroblast-Generated Extracellular Matrix Guides Anastomosis during Wound Healing in an Engineered Lymphatic Skin Flap

Alvis Chiu [1], Wenkai Jia [1], Yumeng Sun [1], Jeremy Goldman [2] and Feng Zhao [1,*]

[1] Stem Cell and Tissue Engineering Lab, Department of Biomedical Engineering, College of Engineering, Texas A&M University, College Station, TX 77843, USA
[2] Vascular Materials Lab, Department of Biomedical Engineering, College of Engineering, Michigan Technological University, Houghton, MI 49931, USA
* Correspondence: fengzhao@tamu.edu

Supplementary Materials:

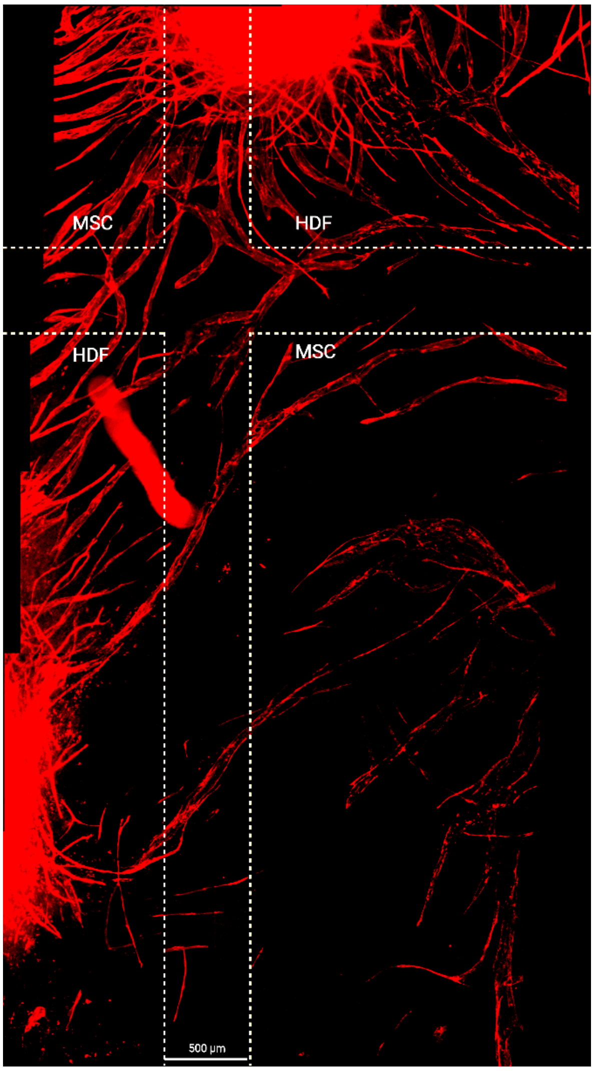

Figure S1. Aggregation in wound healing model at day 10. The 10-hour lymphangiogenic period was extended to 48 hours, resulting in thickened basal cell layer and more complete tube formation. However, basal cell contraction pulled the surface capillaries inward, sometimes over the wound gaps.
